# Characteristics of eligible patients with knee osteoarthritis accepting versus declining participation in a randomised trial investigating the effect of weight loss versus knee arthroplasty to explore generalisability: A cross‐sectional study

**DOI:** 10.1002/ksa.12546

**Published:** 2025-01-05

**Authors:** Saber Muthanna Aljuboori, Robin Christensen, Marius Henriksen, Henning Bliddal, Anders Troelsen, Mikael Boesen, Asbjørn Seenithamby Poulsen, Camilla Toft Nielsen, Kristine Ifigenia Bunyoz, Søren Overgaard

**Affiliations:** ^1^ Department of Orthopedic Surgery and Traumatology Copenhagen University Hospital—Bispebjerg & Frederiksberg Copenhagen Denmark; ^2^ The Parker Institute Copenhagen University Hospital—Bispebjerg & Frederiksberg Frederiksberg Denmark; ^3^ Department of Clinical Medicine, Faculty of Health and Medical Sciences University of Copenhagen Copenhagen Denmark; ^4^ Research Unit of Rheumatology, Department of Clinical Research, University of Southern Denmark Odense University Hospital Odense Denmark; ^5^ Department of Orthopedic Surgery Copenhagen University Hospital—Hvidovre & Amager Hivdovre Denmark; ^6^ Department of Radiology Copenhagen University Hospital—Bispebjerg & Frederiksberg Copenhagen Denmark

**Keywords:** arthroplasty, external validation, generalisability, knee osteoarthritis, weight loss

## Abstract

**Background:**

The INtensive diet versus Knee Arthroplasty (INKA) trial is a randomised trial assessing weight loss as an alternative to knee arthroplasty (KA) in obese patients with severe knee osteoarthritis (OA) awaiting KA (NCT05172843). The external validity of the INKA trial may be hampered if the patients who participate differ from those who decline participation.

**Objective:**

To compare baseline characteristics between patients who enrol in the INKA trial and those who decline participation (i.e., non‐INKA [nINKA] group).

**Methods:**

We applied a cross‐sectional study design, collecting and comparing baseline characteristics among all patients eligible for enrolment in the INKA trial from two clinics in Copenhagen. Imbalance between accepting (INKA) and declining (nINKA) groups was assessed using standardised differences (StdDs). We were prespecified that StdD values < 0.20 would indicate a clinically insignificant imbalance between groups, whereas values > 0.80 indicate incomparability.

**Results:**

Of the 913 patients scheduled for KA, 888 were screened for INKA trial eligibility. Of the 217 eligible patients, 92 (42%) were enroled in the INKA trial, while 37 (17%) participated in the nINKA cross‐sectional sample only. Patients enroled in INKA had on average a less severe Oxford knee score (OKS) of 22.0 (standard deviation = 6.7) compared to declining participants in nINKA with 18.6 (7.2), corresponding to an StdD of 0.50, and an absolute difference of 3.45 (95% confidence interval = 0.64–6.26, *p* = 0.017). A consistent similar pattern was noted across all secondary patient‐reported outcomes applied in the INKA trial.

**Conclusions:**

We observed discrepancies in patient‐reported outcomes, with those who declined enrolment reporting more severe symptoms. These differences, however, were below the minimally important difference between groups for OKS, which is set to 4.84 points.

**Level of Evidence:**

Level II–III cross‐sectional study in a randomised control trial.

**Abbreviations**
95% CI: 95% confidence interval
BMI: body mass index
B‐IPQ: brief illness perception questionnaire
EuroQoL: health outcome and quality of life survey
INKA: INtensive diet versus Knee Arthroplasty
KA: knee arthroplasty
KOOS: Knee injury and Osteoarthritis Outcome Score
MID: minimally important difference
non‐INKA: nINKA
OA: osteoarthritis
OKS: Oxford knee score
OMERACT‐OARSI: Outcome Measures in Rheumatology‐Osteoarthritis Research Society International
OR: odds ratio
PGA: patient's global assessment
QoL: quality of life
RCT: randomised controlled trial
SD: standard deviation
StdD: standardised difference
STROBE: Strengthening the Reporting of Observational Studies in Epidemiology
TKA: total knee replacement
VAS: visual analogue score

## INTRODUCTION

Many risk factors, like increasing age, acute joint injury, joint deformity and overweight, have been linked to osteoarthritis (OA), where overweight is considered to be a main risk factor for developing knee OA [2, 16]. The treatment of knee OA is a multi‐step process, that starts with physiotherapy, weight loss, non‐pharmacological treatment and simple medical treatment as step one, advanced pharmacological treatment as Step 2, a last pharmacological attempt on the third step, and ends with joint replacement for the end stage disease [7]. As obesity is a modifiable risk factor, weight loss is an important part of the treatment strategy for knee OA [7, 31], and weight reduction improves symptoms in patients with knee OA [9, 10, 11]. A cohort study from Australia suggests that the risk of total knee replacement (TKR) can be reduced with weight loss of >7.5% of body weight in adults with overweight or obesity [19]. Furthermore, a weight reduction of 10% before TKR surgery is found to improve the general health‐related outcomes one year after TKR [21].

The INtensive diet versus Knee Arthroplasty (INKA) randomised controlled trial (RCT) aims to assess weight loss as a non‐inferior intervention and/or an alternative to surgical knee arthroplasty (KA) in overweight and obese patients suffering from knee OA (NCT05172843). Recognising the external validity of a trial is crucial for convincing healthcare professionals to contemplate altering the treatment approach. External validity refers to the extent to which the findings and conclusions drawn from the study can be generalised or applied to other populations, settings or conditions beyond the specific context of the trial itself. The Generalisability of trial results is related to whether the randomised trial can be considered pragmatic randomised trial [30]—a clinical trial designed to evaluate the effectiveness of an intervention or treatment strategy in real‐world settings, with the aim of providing evidence that is directly applicable to clinical practice. Systematic reviews have found a tendency for poor representativeness of external validity [8, 14, 20]. We speculated that eligible patients declining inclusion in the INKA trial might have a more severe knee problem, whereas those who are included might be more concerned about having knee surgery. A comparison of the recruited trial participants and the invited participants, who decline trial participation on a range of clinical, paraclinical, and phenotypical variables will enable assessment of the external clinical validity and generalisability of the INKA trial. The assessment of external validity in the INKA trial not only strengthens its findings for clinical practice but also provides valuable insights for future studies where the trial population may systematically differ from the broader target population, an issue often raised in surgical trials [1, 22]. By comparing the characteristics of participants who accept or decline inclusion, we explore aspects of the trial population that extend beyond the predefined inclusion and exclusion criteria. This broader analysis enhances our understanding of patient selection and its impact on the generalisability of RCTs, particularly in pragmatic designs aimed at reflecting real‐world settings [30]. These insights can be taken into consideration in the design of future trials by refining recruitment strategies, thus improving the relevance and applicability of RCT results across diverse populations and clinical environments.

### Objective

Among eligible individuals for the INKA trial, the objective was to compare patients who accepted participation with those who declined, aiming to investigate potential differences between the enroled patients and those who opted not to participate.

## METHODS

### Study design

The INKA trial was designed as a pragmatic, randomised, parallel‐group trial (NCT05172843). It compares the effectiveness of a supervised weight loss programme to surgical KA in obese individuals with knee OA. The present study (the non‐INKA study abbreviated nINKA) was designed as a cross‐sectional study collecting baseline characteristics of patients with knee OA scheduled for KA and eligible for enrolment in the INKA trial. The protocol of the nINKA study has been registered and published along with a statistical analysis plan in clinicaltrials.gov (NCT05215678). Patients declining participation in the INKA trial were asked to participate in the present study. Since the present study design is a cross‐sectional study, we report the results in accordance with the Strengthening the Reporting of Observational Studies in Epidemiology (STROBE) statement for reporting observational studies [15]. Because the study was carried out in hospital departments, it is regarded as ‘public’ in accordance with the Data Protection Agency guidance. The protocol of the present study was approved by the regional Data Protection Agency with the approval number (P‐2022‐48) and all participants provided signed consent to participate in the study.

### Setting

Between January 2022 and September 2023, potential trial participants were identified by orthopaedic surgeons in the orthopaedic outpatient clinics at Copenhagen University Hospitals of Bispebjerg‐Frederiksberg or Amager‐Hvidovre. Those accepting participation in the INKA trial were invited to a subsequent visit, while those who declined INKA were asked to provide baseline characteristics for the nINKA study. An electronic questionnaire was sent to patients accepting nINKA through secured digital post.

### Participants

Eligible patients were adults (i.e., at least 18 years old); had a clinical and radiological diagnosis of knee OA with an indication for primary KA (unicondylar or total arthroplasty); obese (body mass index [BMI] ≥ 30 kg/m^2^) and were motivated for weight‐loss. Patients were excluded if they were scheduled for a revision surgery; planned to have a staged bilateral KA within the observation period; were being operated as sequelae of fractures or due to immune‐inflammatory arthritis; had received injection of medication or substances in the target knee within 3 months prior to participation; systematic treatment with glucocorticoids equivalent to ≥7.5 mg of prednisolone/day; with previous or planned obesity surgery; were unable to understand or read Danish including instructions and questionnaires; or due to any other condition or impairment that in the opinion of the investigator (or his/her delegate) makes a potential participant unsuitable for participation or which obstruct participation.

### Variables

We collected participants demographics (i.e., age, sex, height and body weight), the type of scheduled arthroplasty (total or unicondylar), and patient‐reported measures: The Oxford knee score (OKS) [27], health outcome and quality of life survey (EuroQoL questionnaire) [17], The Knee injury and Osteoarthritis Outcome Score (KOOS)—12 item short form [13, 25], Patient's global assessment (PGA) of impact of the knee in daily life and the brief illness perception questionnaire (B‐IPQ) [24]. Both the INKA and nINKA groups share the same geographical and health insurance characteristics, as all Danish citizens are covered by the Danish health insurance system and both the hospitals cover a homogenous urban geographical area in the capital region.

### Sample size and power considerations

The sample size calculations for the INKA group were based on one‐sided *α* = 0.025, at 90.3% power to detect changes up to 4 points in the OKS assuming a standard deviation of 8 points, this resulted in a required sample size of 86 patients per group (172 in total). To account for attrition (incl. possible ‘cross‐over’) in the intention to treat population it was decided to aim for 200 patients in total. We assumed that half of the patients invited to participate in INKA would decline and accept nINKA participation which would result in 200 patients in the nINKA group. Power and sample size analyses were conducted using ‘proc power’ in SAS (SAS Institute Inc.). For the main analyses presented here, we considered the following ad hoc power calculations to support the validity of this exploratory study on generalisability: Assuming enrolment of 200 participants in each sample (400 patients in the combined nINKA study), we would have 99.9% power to detect a difference between groups as small as four OKS units. In comparison, with the actual sample size of 130 participants allocated in a 3:1 ratio, a prospectively designed study would have 68.1% power to detect a difference of 4 OKS units between groups.

### Statistical methods

We used the term balance diagnostics to describe the methods we use to assess whether the distribution of baseline covariates is similar between the two groups (INKA and nINKA, respectively) [3]. We designed the study to compare estimates of central tendency (mean or medians depending on the empirical data distribution) of continuous variables or the prevalence of dichotomous baseline covariates between INKA and nINKA participants. We report the means and/or medians of continuous variables and the distribution of categorical variables for each of the two groups. These crude comparisons (between INKA=exposure and nINKA=comparator, respectively) enabled an assessment of the comparability of the two groups, which will be indicative of the generalisability of the INKA trial [3].

Standardised differences (StdDs) were used as the main summary measure assessing the balance of baseline characteristics between groups [12]. These StdDs quantify the degree of difference in central tendency between two groups: the exposed group (INKA) and the comparator group (nINKA). The benefit of StdD's lies in their comparability across different variables: They are expressed without units (as unitless values), and thereby allow for a direct comparison of the magnitude of differences between groups, regardless of the scale or measurement units of the variables involved [3]. We prespecified that a StdD of <0.2 could be considered an unimportant imbalance in the baseline covariate, while a StdD ≥0.8 should be considered definitive incomparability (i.e., indicative of poor generalisability) [3, 5]. With the OKS being the primary outcome measure in the INKA trial we added a standard two‐sample test and estimated the difference between means with a 95% confidence interval (95% CI). Given that our primary focus was on exploring generalisability and that we lacked a theoretical basis for testing a series of null hypotheses, we did not find it necessary to adjust for multiple comparisons in this instance. Consequently, the 23 CIs listed in the table are applicable only for interpreting whether the minimal important difference (MID) is included; [12] they were not adjusted for multiplicity and should not be used in place of hypothesis testing.

We also applied a logistic regression model to calculate the propensity of accepting participation in the INKA trial (*Y* = 1) compared to those who declined (*Y* = 0). Finally, for exploratory reasons, we used the Wilcoxon Rank‐sum Test for continuous variables and Fisher's exact test for dichotomous variables to compare the two independent groups (INKA and nINKA, respectively); a two‐sided *p* value <0.05 would usually lead to a rejection of the null hypothesis that the groups are similar. Missing baseline data from one patient in the INKA group was substituted with the group's overall mean value, missing data from nINKA were not imputed and were treated as remaining missing. Data were analysed using the R Statistics version 4.2.1 (www.r-project.org).

## RESULTS

**Figure 1:**
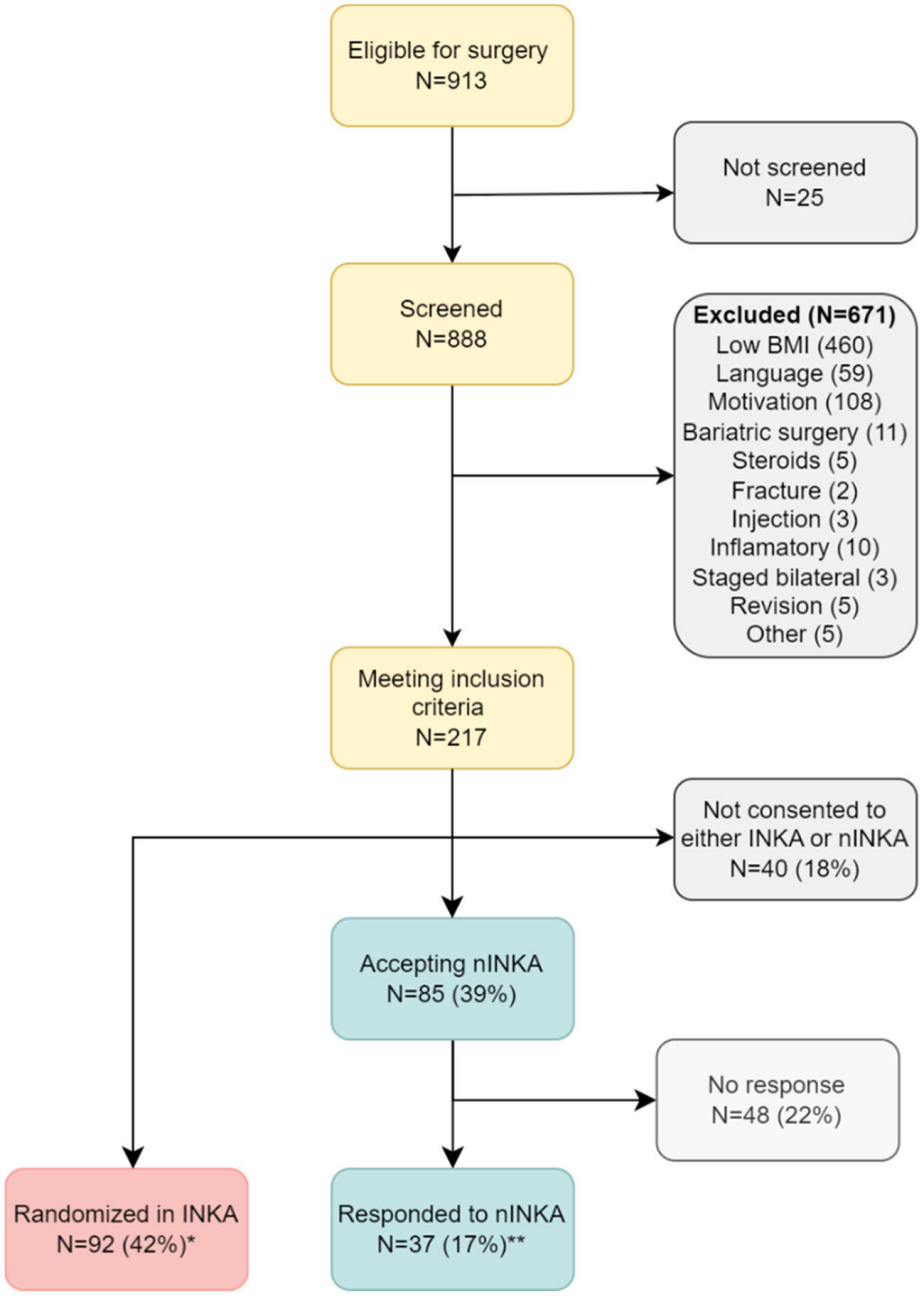
Study flow chart. * One of the patients in the INKA group did not report Oxford knee score (OKS) in the baseline visit. ** Two of nINKA participants had missing data on OKS, other two subjects did not report EuroQoL VAS, PGA and morning pain. INKA, INtensive diet versus Knee Arthroplasty; nINKA, non‐INKA; PGA, patient's global assessment; VAS, visual analogue score.

Patients were recruited between 7 February 2022 and 17 October 2023. In total, 913 patients were scheduled for KA, and from those, 888 were screened for INKA trial eligibility (Figure 1). We excluded 671 patients who did not fulfil the inclusion criteria (Figure 1), leaving 217 eligible for inclusion, 40 refused to participate in both INKA and nINKA. In the end, 92 (42%) participated in INKA, and 85 (39%) accepted to participate in nINKA; however, only 37 of those who accepted participation in nINKA responded to the self‐reported questionnaires.

**Figure 2:**
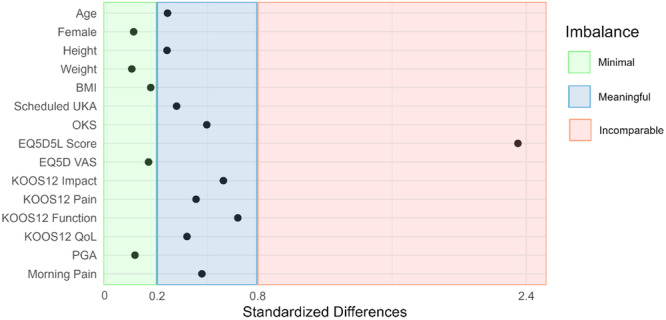
Demonstrating the standardised differences of the means and prevalences and the corresponding predefined imbalance level between INKA and nINKA across the different outcomes; BMI, body mass index; KOOS12, the knee injury and osteoarthritis outcome score 12 item short form; INKA, INtensive diet versus Knee Arthroplasty; nINKA, non‐INKA; OKS, Oxford knee score; PGA, patient's global assessment; QoL, quality of life; UKA, unicondylar knee arthroplasty; VAS, visual analogue score.

**Figure 3:**
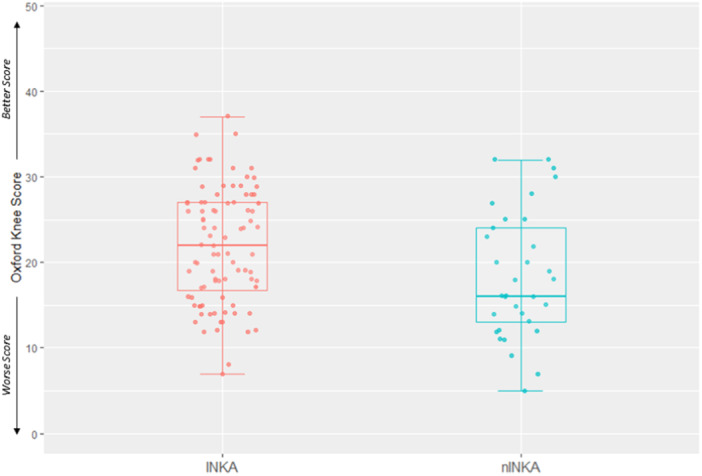
Individual Oxford knee score (OKS) for both INKA and nINKA groups. The dots represent the actual data points and the distribution of the data set along the single OKS axis. The boxplots provide a visual representation of key statistical measures: the middle line represents the median, while the box itself spans from the first quartile (25th percentile) to the third quartile (75th percentile). Additionally, whiskers extend from the box to the furthest data points within 1.5 times the interquartile range. INKA, INtensive diet versus Knee Arthroplasty; nINKA, non‐INKA.

For the primary outcome measure, eligible patients who accepted participation in the INKA trial had on average a less severe OKS of 22.0 (standard deviation [SD] = 6.7) compared to participants in nINKA with 18.6 (SD = 7.2) with an StdD of 0.50; see Table 1 and Figure 2. Individual data points and the distribution of the data set along the single OKS axis showing the imbalance between the two groups are illustrated in Figure 3. It also displays the median and quartiles, corresponding to an absolute difference between means of 3.45 (95% CI = 0.64–6.26; *p* = 0.017). For all the secondary patient‐reported outcomes applied in the INKA trial, a consistent pattern was noted across all measurements, showing that these scores were less severely affected in the participants accepting trial participation compared with those who declined (Table 1).

**Table 1:** Baseline characteristics of participants enroled in the randomised controlled trial (INKA) and the non‐INKA (nINKA) cross‐sectional study.

Characteristic	INKA (*n* = 92)	nINKA (*n* = 37)	StdD	OR^b^ [95% CI]
Age, years	67.5 (8.4)	65.4 (7.4)	0.26	1.03 [0.98–1.08]
Female sex, *n* (%)	57 (62)	24 (65)	0.06	0.88 [0.40–1.95]
Height, cm	169 (10)	171 (9)	0.26	0.97 [0.93–1.01]
Weight, kg	106 (16.8)	107 (16)	0.05	1.00 [0.97–1.02]
BMI, kg/m^2^	36.9 (4.3)	36.2 (4.2)	0.16	1.04 [0.95–1.14]
Scheduled UKA, *n* (%)	59 (64.1)	18 (48.6)	0.32	1.89 [0.87–4.09]
Oxford knee score, 0–48^a^	22 (6.73)	18.6 (7.18)	0.50	1.08 [1.01–1.14]
EuroQoL Index, median [IQR], −0.757 to 1.000	0.72 [0.39–0.81]	0.20 [0.05–0.33]	2.35	22.7 [6.24–82.4]
EuroQoL VAS, 0–100^a^	52 (24.8)	48.5 (21.8)	0.15	1.01 [0.99–1.02]
*KOOS12 domains*				
Impact, 0–100	39 (12.8)	31.6 (12)	0.60	1.05 [1.01–1.08]
Pain, 0–100	39.2 (14.2)	33.3 (13.3)	0.43	1.03 [1.00–1.06]
Function, 0–100	45.2 (16.1)	34 (16.9)	0.68	1.04 [1.02–1.07]
Quality of life, 0–100	32.4 (13.7)	27.5 (11.6)	0.38	1.03 [1.00–1.06]
*B‐IPQ*				
Item 1, 0–3	2.6 (0.54)	2.7 (0.46)	0.21	0.66 [0.30–1.44]
Item 2, 0–3	2.37 (0.72)	2.27 (0.73)	0.14	1.21 [0.72–2.03]
Item 3, median [IQR], 0–3	1.00 [1.00, 2.00]	1.00 [0.00,1.00]	0.00	1.64 [1.01–2.67]
Item 4, 0–3	2.71 (0.48)	2.51 (1.02)	0.24	1.47 [0.87–2.50]
Item 5, 0–3	2.53 (0.54)	2.62 (0.49)	0.17	0.72 [0.34–1.52]
Item 6, 0–3	2.57 (0.60)	2.68 (0.53)	0.20	0.70 [0.35–1.42]
Item 7, 0–3	2.45 (0.69)	2.38 (0.76)	0.09	1.14 [0.67–1.95]
Item 8, 0–3	1.84 (0.91)	1.97 (0.83)	0.16	0.84 [0.54–1.30]
PGA, 0–100	74.1 (17.7)	75.2 (14.3)	0.07	1.00 [0.97–1.02]
Morning pain, 0–100	55.2 (26.4)	66.2 (20.4)	0.47	0.98 [0.96–1.00]

*Note*: Plus‐minus values are means (±standard deviation) unless otherwise indicated.

Abbreviations: 95% CI, 95% confidence interval; B‐IPQ, brief illness perception questionnaire; BMI, body mass index; KOOS12, the knee injury and osteoarthritis outcome score 12 item short form; INKA, INtensive diet versus Knee Arthroplasty; IQR, interquartile range; nINKA, non‐INKA; OR, odds ratio; PGA, patient's global assessment; StdD, standardised difference; UKA, unicondylar knee arthroplasty.

^a^ In nINKA, two participants had missing data on Oxford knee score, EuroQoL VAS, PGA and morning pain.

^b^ OR with 95% CIs reports on all univariate logistic regression models reflecting the impact on a propensity score.

Among eligible patients, those who were more likely to accept participation in a weight loss trial were those with less severe symptoms at referral regarding KA (Table 1). Based on the propensity to accept participation in the INKA trial, the analysis indicates that patients with higher OKS at referral, thus less symptoms, were more inclined to accept to participate in a weight loss trial (odds ratio [OR] = 1.08, 95% CI = 1.01–1.14).

## DISCUSSION

The study aimed to compare baseline characteristics among patients who accepted enrolment in the INKA trial versus those who declined participation. We showed that the patients who declined to participate in the nINKA group had worse symptoms in OKS, EuroQoL Index, KOOS12 pain, function, quality of life (QoL) domain, B‐IPQ, and morning pain. This could challenge the external validity of the INKA trial. The between‐group difference in our study did, however, not reach the minimally important difference (MID) estimate applicable for group comparisons in RCTs, which is suggested to be 4.84 OKS points [4].

The external validity of RCTs is a major concern as it affects the applicability of RCTs' results, and study designs that take external validation into consideration are sparse in the literature [26]. The importance of external validation is confirmed by the consistent systematic difference between recruited and non‐recruited groups [26]. The results of our study that the declining patients had worse symptoms at baseline suggest that the possible outcome from intensive weight loss may be less valued by patients with worse patient‐reported measures. It may be explained by several reasons: [18] First, due to advanced disease and concerns about potential delays in surgery. Second, because of the possibility that these patients had experienced previous weight loss treatment failures, leading to expectations of poor long‐term outcomes from weight loss. Third, logistical challenges associated with participation and expressed fear of inferior outcomes compared to surgical treatments. We do not believe that the communication of the study information to the patients had any influence, as it should not have been different due to standardised patient information. However, we cannot exclude a communication factor that might have influenced the patient's choice due to severe symptoms.

Studies have shown that the recruitment by healthcare professionals can influence the patients’ choice to participate in trials as in a randomised trial comparing surgical vs. non‐surgical treatment for type‐B ankle fractures showing that patients declining to participate in the trial tended to prefer the non‐surgical treatment and that they were influenced by the healthcare professional [23]. These findings come parallel with findings from other studies suggesting that the individual treatment preference of the healthcare professional informing the patient about the trial would influence the patient choice, that is, patients that the recruiting healthcare professional doom not to benefit from the intervention would less likely be included [18]. As the recruiting healthcare professional in the INKA trial was a knee surgeon, it could be possible that the personal influence of the healthcare professional affected the decision of patients with worse symptoms to go directly to surgery and decline participation in INKA. Finally, it is possible that an explanation of the differences between the two groups is found in patients' expectations of trial outcomes and personal influence factors.

In a related trial looking into the feasibility of a preoperative package of care for OA for patients on the waiting list for receiving KA. Out of 223 screened patients, 163 (73%) were excluded from the study, of which 67 (30%) due to refusal to participate. Notably, unwillingness to comply with the study protocol was an exclusion criterion, further narrowing the study population [28]. This may result in low external validity. This was evaluated in our study by comparing the baseline characteristics of accepting versus declining patients which made it possible to identify the population that the study results apply beyond what the eligibility criteria can present. Examining the eligibility criteria of studies helps in determining the population that the results can apply to; this however does not take into consideration the characteristics of the eligible group declining to participate in trials. Many measures can be taken to increase the recruitment rate in trials [6], however cannot secure an inclusion rate that can guarantee generalisable real‐world results.

As a consequence of conducting numerous statistical tests, we acknowledge the potential for Type I errors resulting from multiple comparisons. While we did not adjust for these in this exploratory study due to a lack of theoretical hypotheses suggesting significant differences, it is important to emphasise that the confidence intervals were not adjusted for multiplicity and should not be interpreted as substitutes for hypothesis testing. The observed differences in baseline characteristics between the INKA trial participants and those who declined participation have significant implications for interpreting the trial results. Specifically, the worse symptom profiles of the nINKA group suggest that the outcomes achieved in the INKA trial may not fully reflect the experiences of a broader population with knee OA, particularly those with more severe symptoms who may prioritise surgical intervention over non‐surgical treatments. Clinically, this raises concerns about the potential for selection bias in trials that do not account for these differences. Adopting a similar approach can enhance the generalisability of the trial beyond the conclusions drawn from the inclusion and exclusion criteria by looking into eligible patients who were not included. Future designs should explore the motivations behind patient decisions to decline enrolment and incorporate qualitative assessments of patient preferences and expectations regarding treatment outcomes. This would not only improve our understanding of trial generalisability but also promote the development of more inclusive approaches in clinical research.

### Strengths

Our study included patients from two surgical centres in Copenhagen, with a predefined protocol and statistical analysis plan (NCT05215678). We used a rigorous assessment of patient‐reported outcomes with domains covering recommendations from the Outcome Measures in Rheumatology‐Osteoarthritis Research Society International (OMERACT‐OARSI) (pain, physical function, QoL and patient global assessment of target joint) to explore in depth the possible imbalance between consenters and non‐consenters to the INKA trial [29]. The use of StdDs is, unlike inferential statistics and statistical tests of hypothesis, not (directly) influenced by sample size. It is rather dependent on the visually appearing difference captured by the means taking into consideration the variance, making it intuitive and easy to interpret; that is, several StdDs can be compared directly, at least for descriptive purposes, even when they originate from different sample sizes [3].

### Limitations

A concern is the low response rate from the participants in the nINKA group, representing only 30% of the total non‐consenters to the INKA trial. While we do not believe this low response rate critically undermines our primary conclusions due to the minimal imbalance between the two groups in age, sex distribution, and BMI, we acknowledge that it limits the precision and generalisability of our findings. This low representation raises the possibility that the nINKA population may not fully represent the target population, potentially challenging the interpretation of this study. Future studies should consider strategies to enhance response rates, such as collecting participants' responses during in‐clinic visits rather than relying on electronic forms. Additionally, incorporating qualitative approaches to explore reasons for non‐participation could provide valuable insights into the factors influencing these decisions.

Five of the 671 excluded patients were excluded due to other causes as deemed by the recruiting surgeon. This may be a source of selection bias as these could be eligible patients that were subjectively not selected, however the impact on the results is minimum as they represent less than 1% of the excluded population. Another limitation could be some baseline characteristics that we were unable to measure, such as socioeconomic status, smoking and alcohol use. However, we do not believe the absence of these unmeasured factors will affect our findings in terms of the effect size of the measured outcomes; instead, they may reveal further imbalances between the two groups.

## CONCLUSION

We found minimal imbalances in demographics and anthropometric measures, while there were discrepancies in patient‐reported outcomes, with those who declined enrolment reporting more severe symptoms; these differences did however exclude the MID for OKS.

Although caution is needed when interpreting these findings due to the possibly limited representation of the nINKA group within the total cohort of declining participants, these disparities may suggest potential constraints on the generalisability of the INKA trial.

## AUTHOR CONTRIBUTIONS

Study conception and design: Saber Muthanna Aljuboori, Robin Christensen and Søren Overgaard. Recruitment of patients: Saber Muthanna Aljuboori and Kristine Ifigenia Bunyoz. Acquisition of data: Saber Muthanna Aljuboori and Marius Henriksen. Analysis and interpretation of data: Saber Muthanna Aljuboori, Robin Christensen and Søren Overgaard. Drafting the article or revising it critically for important intellectual content: All authors. Final approval of the article: All authors.

## CONFLICT OF INTEREST STATEMENT

The authors declare no conflict of interest.

## ETHICS STATEMENT

This study only involved questionnaires and medical records and was hence exempt from approval from The Health Research Ethics Committee and was implemented without permission from the Ethics Committee according to Danish legislation (Heath Research Ethics Committee Act § 1, paragraph 1). A written, informed consent was received from all included patients. The study was notified to the local Data Protection Agency (approval number: P‐2022‐48).

## Data Availability

The data that support the findings of this study are available from the corresponding author upon reasonable request.
